# Priorities for a gender‐sensitive sexually transmitted infections and human immunodeficiency virus (STIs/HIV) services: An exploratory mixed methods study

**DOI:** 10.1002/hsr2.553

**Published:** 2022-03-10

**Authors:** Masoumeh Simbar, Fatemeh Rahmanian, Soheila Nazarpour, Ali Ramezankhani, Farid Zayeri

**Affiliations:** ^1^ Department of Midwifery and Reproductive Health, School of Nursing and Midwifery, Midwifery and Reproductive Health Research Center Shahid Beheshti University of Medical Sciences Tehran Iran; ^2^ Department of Midwifery, School of Nursing and Midwifery Shiraz University of Medical Sciences Shiraz Iran; ^3^ Department of Midwifery, Chalous Branch Islamic Azad University Chalous Iran; ^4^ Department of Public Health, School of Public Health and Safety Shahid Beheshti University of Medical Sciences Tehran Iran; ^5^ Department of Biostatistics Shahid Beheshti University of Medical Sciences Tehran Iran

**Keywords:** gender, health service assessment, reproductive health services, STIs/HIV prevention

## Abstract

**Background and Aim:**

Providing gender‐sensitive health services is emphasized by the World Health Organization. This study aimed to assess and prioritize the needs for the gender‐sensitive sexually transmitted infections/human immunodeficiency viruses (STIs/HIV) prevention services by a valid and reliable questionnaire.

**Methods:**

This was an exploratory mixed methods sequential study in Shiraz Iran 2019. The first phase was a qualitative study on 37 providers and managers of the services who were recruited using the purposive and then snowball sampling method. In the second phase, following the content analysis of the qualitative data and a review of related literature, a questionnaire was developed and its psychometric properties were evaluated. Then, in the third phase, the questionnaire was used to assess and prioritize the needs through a quantitative descriptive cross‐sectional study on all 290 providers of STI/HIV prevention services affiliated with Shiraz University of Medical Sciences.

**Results:**

The finding of the qualitative phase showed gender‐sensitive STI/HIV prevention services should provide gender‐sensitive care and education by the trained personnel and manages with appropriate facilities and equipment. Providing these services also requires supportive policies, intersectoral cooperation, and community capacitation. In the second phase, a questionnaire was developed with 63 items. Assessment of psychometric properties of the questionnaire demonstrated the scale content validity index and ratio (S‐CVI = 0.98 and S‐CVR = 0.87, respectively), as well as the reliability of the questionnaire (internal consistency = 0.972 and intracluster correlation coefficient = 0.910). Results of the third descriptive phase of the study demonstrated the highest priorities for gender‐sensitive education (92.01 ± 11.76%) and care services (92.11 ± 12.04%), respectively.

**Conclusions:**

To improve the quality of the services, a gender‐based education and care process, as well as a gender‐sensitive structure, including gender‐sensitive personnel, facilities, and management are necessary. Recognizing and meeting the needs for gender‐sensitive services will improve the quality of the services.

## 1 INTRODUCTION

Gender norms and roles can influence individuals' mental, physical, social health, and well‐being.^1^ Gender norms can impact women's and men's health through different health‐related behaviors and access to care.^2^ There is a growing trend of documents showing that gender norms and behaviors are effective on the pattern of spreading sexually transmitted infections/human immunodeficiency virus (STIs/HIV) among men and women.^3^ Studies show a higher frequency of STIs and HIV and more vulnerability of women comparing to men.^4^ A review on 130 studies during 2009–2016 reports on the prevalence of STIs, including chlamydia, gonorrhea, trichomoniasis, and syphilis showed the prevalence of 3.8%, 0.9%, 5.3%, and 0.5% in women, respectively; and the prevalence of 2.7%, 0.7%, 0.6%, and 0.5% among men, correspondingly.^5^

Restricted gender norms and the low social status of women make them vulnerable to STIs/HIV. This problem may arise due to their very low authority for decision‐making in sexual relationships.^6^ Sometimes, women hide STIs as they concern to lose their partner, or they do not seek ways to protect themselves in the event of a partner's STIs infection.^7^ Also, male gender roles may predispose them to STIs. Early initiation of sexual relationships and having multiple sex partners increase male risk of STIs.^8^, ^9^ Furthermore, men would not usually seek counseling and treatment services for STIs/HIV and acquired immunodeficiency syndrome (AIDS) because of a myth that “seeking care is a sign of weakness” and it is not masculine behavior in their gender norms.^5^, ^10^ Therefore, providing gender‐based counseling and care services seems to be necessary for STIs/HIV care services.

Gender is a fundamental factor that shapes health systems and outcomes.^11^ Gender norms are among the most important effective factors on STIs/HIV incidence and care services.^12^ Hence recognizing the behaviors arising from gender norms affecting STIs/HIV prevention behaviors and services seems to be necessary.

Nowadays, providing gender‐sensitive health services is emphasized by the World Health Organization.^13^, ^14^ Gender‐sensitive health services mean that health authorities have the knowledge and are able to perceive existing gender differences and to integrate these into their decision‐making and actions.^15^ Therefore, gender‐based special needs in any community and culture should be known and considered in providing STIs/HIV prevention services.^16^ The needs assessment requires a valid and reliable tool. Therefore, developing valid and reliable questionnaires to assess the real needs for providing gender‐sensitive services helps health care managers to improve the quality of the services.^17^ To our knowledge either there are limited studies to assess needs for gender‐based reproductive health services^17^, ^18^ or no tool to assess needs for providing STIs/HIV prevention services.

There are very few studies on the gender‐based special needs for STI/HIV prevention services. Lichtenstein et al.^19^ showed men's concern about stigmatization as the main barrier for seeking STI prevention and treatment services. Garcia et al.^20^ stated that stigma causes many people to avoid seeking STI‐related services because of experiences, such as discrimination, indifference, and overt hostility in the health care setting. They mentioned to a world‐wide barrier to a full range of reproductive health services such as restricted access to STI testing, criminalization of sexual behaviors associated with STI transmission, for example, commercial sex work or same‐sex sexual relationships. A study in Nigeria demonstrated that a greater proportion of males than of females had sought treatment for their STIs (64% vs. 48%). Females had lower odds than males of having sought STI treatment (odds ratio: 0.6).^21^ Rahmanian et al.^22^ in Iran used a valid and reliable questionnaire to assess the needs of gender‐sensitive adolescents reproductive health services (ANQ‐GSARHS), indicating the priorities for providing gender‐sensitive services for adolescents, such as providing contraceptives for female adolescents, educating female adolescents about STIs, counseling male adolescents about confronting with peer pressure, employment of trained male providers for male adolescents' reproductive health services, and improving knowledge of providers about adolescents' reproductive health. However, to our knowledge, there is no study to understand the gender‐based needs of STIs/HIV prevention services. Besides, very few tools are available to assess gender sensitivity in STIa services,^23^ male participation in perinatal care services,^24^ and reproductive health services.^25^ The most comprehensive questionnaire to assess gender sensitivity in reproductive health services is available at the level of staff and facilities.^26^

Therefore, considering the daily incidence of more than 1 million (STIs) cases worldwide^27^; 26.4 million cases of four curable STIs (*Chlamydia trachomatis*, *Neisseria gonorrhoeae*, *syphilis*, and Trichomonas vaginalis) in the Eastern Mediterranean region^28^; and respecting to 59,531 estimated number of people with HIV in Iran in 2021^29^ and also growing spread of STIs and HIV in Iran,^30^ as well as the importance of gender‐specific needs assessment in STIs/HIV health services with a valid and reliable tool, this exploratory mixed sequential study aimed to assess and prioritize the needs of gender‐sensitive STIs/HIV prevention services, by a valid and reliable questionnaire.

## 2 METHODS

### 2.1 Design of the study

This was a mixed‐method exploratory sequential qualitative–quantitative study. The exploratory sequential study is used when the researcher is interested in following up qualitative findings with quantitative analysis. This two‐phase approach is particularly useful for a researcher interested in developing a new instrument or treatment protocol.^31^ Therefore, the first phase of the present study was a qualitative study to produce codes and themes. These codes were used for the concept definition and item generation of the questionnaire. In the second phase, the questionnaire's psychometric properties were assessed. Thereafter, in the third phase, the questionnaire was used for a descriptive cross‐sectional study to assess and prioritize the needs for gender‐sensitive STIs/HIV prevention services.^31^ Therefore, the method section is presented in these three abovementioned phases, (1) qualitative phase, (2) tool development phase, and (3) quantitative phase.

### 2.2 Phase 1: The qualitative phase of the study

#### 2.2.1 Design

This phase of the study was a qualitative study with a content analysis approach. The codes extracted from the qualitative phase were used for item generation for the questionnaire. For developing the questionnaire, the steps described by Waltz et al.^32^ were used. Respecting the first step for the questionnaire development, the qualitative phase was performed to explain needs for gender‐sensitive STIs/HIV prevention services and the extracted codes were used for the items generation.

#### 2.2.2 Study settings

The research interviews were conducted in primary health care centers, voluntary counseling and testing services, and hospitals affiliated with Shiraz University of Medical Sciences (SUMS), as well as the relevant STIs/HIV prevention headquarters in Shiraz, and the Ministry of Health in Tehran in Iran. The participants were all interviewed in their workplace

#### 2.2.3 Participants

Participants were STIs/HIV care providers and managers or policymakers.

#### 2.2.4 Eligibility criteria

*The inclusion criteria* for participation were at least 2 years of working experience as a provider, manager, or policymaker of STIs/HIV prevention services.

#### 2.2.5 Sampling method

The participants were selected using the purposive and snowball sampling method. Recruitment continued until data saturation was reached.

#### 2.2.6 Outcome measures

Explaining the need for gender‐sensitive STII/HIV prevention services.

#### 2.2.7 Tool for data collection

Data were collected using in‐depth interviews using a semi‐structured questionnaire. The guide questions for the interview were: “How do you perceive the concept of gender‐sensitive STIs/HIV prevention services”; “what is your perception about the gender roles that may affect sexual health”; and “what is necessary for a gender‐sensitive STIs/HIV services?” and “how these services could be provided?” A demographic questionnaire was also used for data collection.

#### 2.2.8 Study procedure

Data were collected using a deep face‐to‐face individual interview by using the semi‐structured interviews and continued until data saturation; that is, when no new code of data was added to the study. The interviews were conducted by the second author Dr. Rahmanian, who is an assistant professor in the Department of Midwifery and Reproductive Health at SUMS. After being introduced to the interviewer, the participants were informed about the goals of the study and promised the confidentiality of their personal information. Also, field notes were made during and after the interview. The interviews were performed after two pilot interviews. The average duration of interviews was about 60–90 min. All interviews were audiotaped, transcribed verbatim, and analyzed consecutively.

#### 2.2.9 Data rigor and trustworthiness

To assess the trustworthiness of data, four criteria of Lincoln and Guba's^33^ were used, including credibility, dependability, confirmability, and transferability. To increase credibility, adequate time was allocated to data collection and frequent reviewing of the data. In addition, integration of data collection methods, that is, individual interviews and observations increased the credibility of the data. The participants (the service providers) were observed at the STIs/HIV prevention services and field notes were taken. Field notes are a qualitative approach, most often used in ethnography. Field notes are written observations recorded during or immediately following participant observations in the field and are considered critical to understanding phenomena encountered in the field.^34^

Some codes extracted from interviews were reviewed by four participants, including two managers and also two service providers, who had not participated in the study to ensure that the results accurately depicted the participants' experiences and perceptions. To confirm the dependability of the data, code‐recode and external checking were used. To assess the confirmability of the data, the researchers abandoned all their assumptions and thoughts and carefully documented all the research steps, and allowed external auditors to investigate all the steps. To ensure transferability and comprehensiveness, a clear explanation of the methods of collecting and analyzing the data was presented along with examples of the statements made by the participants.

#### 2.2.10 Data analysis

The data were analyzed by using MAXQUDA10 and a conventional content analysis based on the criteria proposed by Graneheim and Lundman.^35^ After transcribing, the recorded interviews, the transcripts were carefully reviewed by the researcher two to three times to achieve an accurate understanding of the interview contents. The text was divided into meaning units, meaning units were condensed while preserving the meaning and labeled with codes. Similar codes were then categorized into subcategories, and the subcategories were classified into a category based on common properties. The latent content of the similar categories was eventually formulated as a theme.

#### 2.2.11 Ethical considerations

Before conducting the interviews, the researcher briefed the participants on the objectives of the study and assured them that their responses will be confidential. Informed written and verbal consent was also obtained from the participants for participating in the study and recording the interviews.

### 2.3 Phase 2: The questionnaire development and assessment of the psychometric properties

The method of this phase of the study is described in two parts: (1) the questionnaire development and (2) the assessment of psychometric properties of the questionnaire.

#### 2.3.1 The questionnaire development

2.3.1.1

*Design*: To develop the questionnaire the deductive‐inductive approach was conducted. In other words, the items were generated from the extracted codes from the qualitative, and then were completed by the extracted items from the literature review. Also, Waltz et al.'s^32^ four steps of a questionnaire development were used.

The qualitative phase was performed using the conventional content analysis method to explain the concept and dimensions of gender‐sensitive STI/HIV prevention services. The primary pool of items and subscales of the questionnaire were respectively generated from codes and categories extracted from the data analysis of the qualitative phase. A few items were also added to the pool after a literature review.

2.3.1.2

*The literature review*: To ensure that the items and construct definition aligns with relevant prior research and to identify existing survey scales or items that might be used, an extensive literature review was conducted.

2.3.1.3

*Search strategy*: The preferred reporting items for systematic reviews^36^ was used to identify and articulate the needs for the STIs/HIV services. We used appropriate operators, such as AND and OR, and a combination of search strategies for each database. We conducted a comprehensive search using the keywords “STI,” “HIV,” “AIDS,” “prevention,” “Care,” “Service,” “Gender,” and “questionnaire” in Scopus, PubMed, Science Direct, Google Scholar, SID, World Health Organization, International Confederation of Planned Parenthood, United Nation Population fund websites, and Magiran databases.

2.3.1.4

*Eligibility criteria*: The inclusion criteria were Persian or English sources, the articles published between 1990 and 2019 in the abovementioned databases.

Some appropriate items were recognized in the review and added to the primary pool of items. In this way, the initial questionnaire was prepared for evaluating the psychometric properties.

#### 2.3.2 Psychometric properties of the questionnaire

The questionnaire was assessed regarding its validity and reliability as below:

2.3.2.1

*Validity of the questionnaire*: Face validity and content validity as the theoretical and representational validity of the questionnaire was evaluated by qualitative and quantitative methods. The validity of a questionnaire can be established using a panel of experts that explore theoretical constructs. This form of validity exploits how well the idea of a theoretical construct is represented in an operational measure. This is called translational or representational validity.^37^

2.3.2.2

*Face validity*: For qualitative face validity assessment, 15 providers of STIs/HIV prevention services were asked about the items' difficulty, irrelevancy, and ambiguity.^32^ Next, for quantitative face validity assessment, they were asked to specify the importance of the items on a 5‐point scale. Then the impact scores were calculated using the following formula: “impact score = importance × frequency.” Afterward, the impact score of each item was calculated and evaluated by the cut‐off point of >1.5.^38^, ^39^ So, the items with the impact score ≥1.5 was considered appropriate.

2.3.2.3

*Content validity*: For qualitative content validity assessment, 10 experts in reproductive health, midwifery, and nursing were asked to provide feedback on the questionnaire regarding grammar, appropriate word use, and appropriate placement of phrases. Then, for quantitative content validity assessment, the content validity ratio (CVR) and content validity index (CVI) were measured.^40^

2.3.2.4

*Content validity ratio*: To ensure the selection of the most important items, CVR was calculated for each item. The experts signified their opinions by assigning each item scores of 1–3, which correspond to “not necessary,” “useful but not essential,” and “essential,” respectively. The scores were then calculated using the following formula: CVR = (Ne – *N*/2)/(*N*/2), where Ne is the number of experts indicating an item as “essential” and *N* is the total number of experts. The accepted value was determined based on Lawshe's table and the number of experts.^41^ The opinions of the 10 experts were referred to in evaluating the CVR with 0.62 regarded as acceptable.^41^

2.3.2.5

*Content validity index*: This index was calculated based on Waltz and Bausell's^40^ criteria to ensure that the items of the questionnaire are appropriately designed to measure content. The expert evaluation was focused on relevance, clarity, and simplicity and was expressed using a 4‐point Likert scale (scores 1–4, respectively).

The CVI score of each item was computed as the number of experts giving a rating of 3 or 4 to the relevancy, clarity, and simplicity of each item, divided by the total number of experts. Based on this index, an entire statement was initially measured in terms of relevance, after which its acceptability was determined according to the following criteria: CVI > 0.79, the item is relevant, between 0.70 and 0.79, the item needs revisions, and if the value is below 0.70 the item is eliminated.^42^ Scale‐level content validity index (S‐CVI) and scale‐level content validity ratio (S‐CVR) were also computed by calculating the mean of CVI and CVR values. S‐CVI 0.9% were considered as the acceptable validity index of the questionnaire.^42^

2.3.3

*Reliability of the questionnaire*: To assess the reliability of the questionnaire, the internal consistency, as well as the stability of the questionnaire, was measured.

2.3.3.1

*Internal consistency*: Cronbach's coefficient *α* was calculated to examine the internal consistency of the subscales and the entire instrument. Values above 0.79 are considered acceptable in a descriptive study.^43^

2.3.3.2

*Stability*: The stability was also assessed by the test–retest method and through the completion of the questionnaires by 15 care providers within a 2‐week interval. Intraclass correlation coefficient (ICC) was also calculated to assess the stability of the questionnaire. If the ICC is above 0.7, the stability was considered appropriate.^44^

2.3.3.3

*Describing the questionnaire and the scoring system*: The final version of the questionnaire was a valid and reliable questionnaire that was used to measure needs for gender‐sensitive STIs/HIV prevention services with three‐level scales from “not at all” to “completely” important, which were scored from 0 to 2. The total score and the scores for the subscales of the questionnaire were calculated and converted to 0–100. High scores show more important needs for the gender‐sensitive STIs/HIV prevention services.

#### 2.3.4 Outcome measures

CVI, CVR, *α* Cronbach's, ICC of the questionnaire were the outcome measures in this phase of the study.

### 2.4 Phase 3: The quantitative phase of the study “needs assessment for a gender‐sensitive STIs/HIV prevention services”

#### 2.4.1 Design of the study

This was a descriptive cross‐sectional study in Shiraz‐Iran 2019.

#### 2.4.2 Subjects of the study

All 290 providers of STIs/HIV prevention service providers participated in the study.

#### 2.4.3 Inclusion criteria

The eligibility criteria for participation were at least 2 years of work experience in reproductive health care services, including STIs/HIV prevention care and counseling.

#### 2.4.4 The setting of the study

All 37 health centers affiliated with SUMS and nine hospitals in Shiraz‐Iran.

#### 2.4.5 Sampling

All 290 STIs/HIV prevention care providers. So, the subjects of the study were recruited using the survey convenience method of sampling.

#### 2.4.6 Tool for data collection

The tools for data collection were (1) a demographic information questionnaire and; (2) a valid and reliable questionnaire that was developed in the second phase of the study (gender‐sensitive STIs/HIV prevention services [GSPS]). The validity and reliability of the questionnaire are described in the second phase of the study.

#### 2.4.7 Outcome measures

The outcome measure in this phase of the study was the score of the needs for gender‐sensitive STIs/HIV prevention services. The needs were assessed by GSPS. High scores show more important needs for the gender‐sensitive STIs/HIV prevention services.

#### 2.4.8 Data analysis

**Figure 1:**
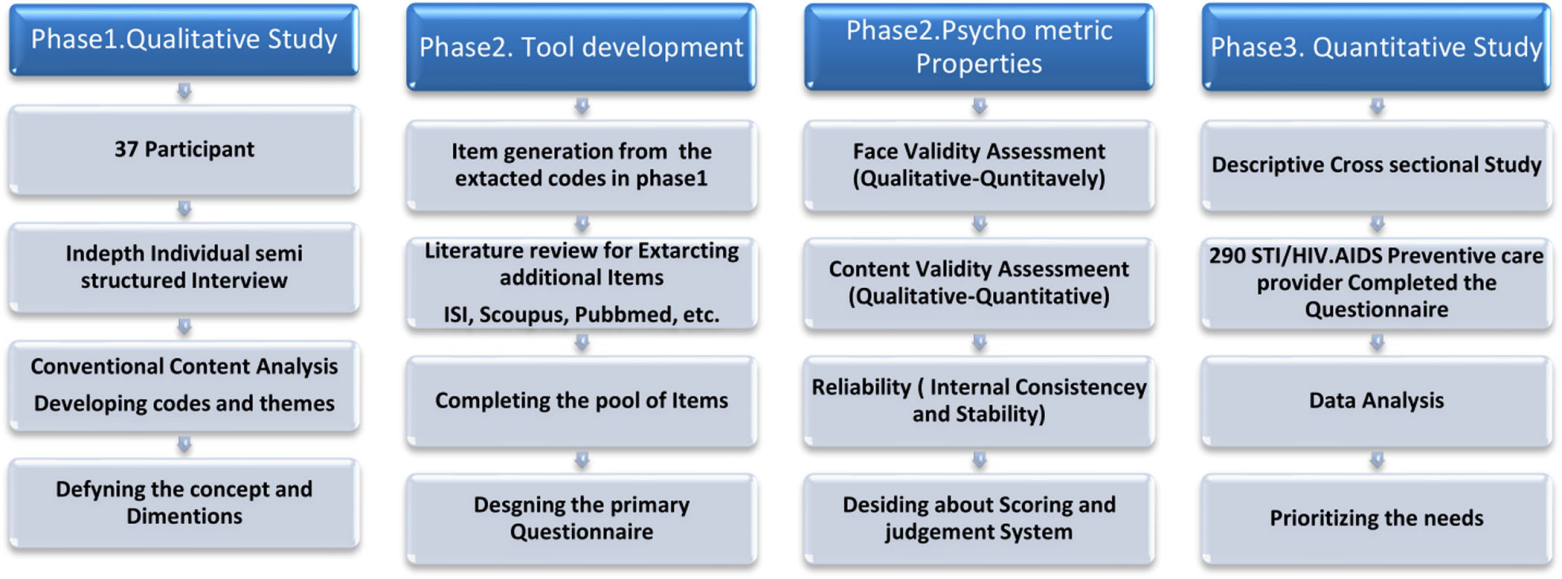
Procedure of the exploratory mixed sequential qualitative–quantitative study to assess needs for the gender‐sensitive STIs/HIV prevention services. AIDS, acquired immunodeficiency syndrome; STI/HIV, sexually transmitted infections/human immunodeficiency virus

Data were analyzed by SPSS V‐22. *p* < 0.05 was considered significant. Figure 1 shows the procedure of the study.

### 2.5 Ethical considerations

Ethics were considered by describing the aims and procedure of the study for the participants. Written consent was obtained from all participants of the study. The confidentiality of information was guaranteed, as the name and personal information were not mentioned in tapes, transcripts, and the questionnaires. Permission was given by authorities in Shiraz and Tehran. The study was approved by the Ethical Committee of Shahid Beheshti University of Medical Sciences.

## 3 RESULTS

### 3.1 Phase 1: The qualitative phase of the study

**Figure 2:**
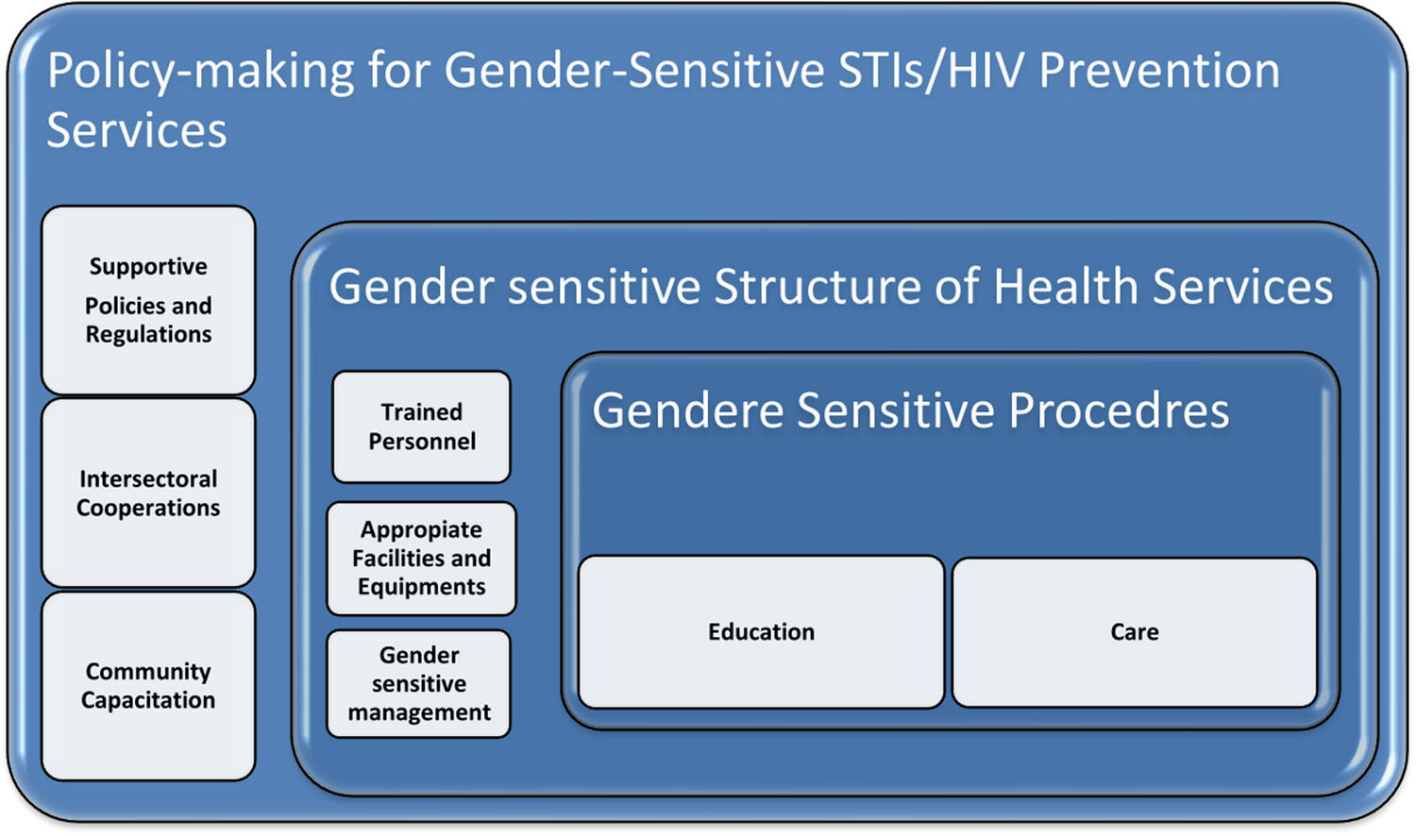
A schematic diagram of concept and dimensions of gender‐sensitive STIs/HIV prevention and care services. STIs/HIV, sexually transmitted infections/human immunodeficiency virus

In the qualitative phase, 37 participants (14 general practitioners, 13 midwives, 6 public health providers, 3 nurses, and a previous Minister of Health) were interviewed. They were also 13 men and 24 women. The average age of participants was 43.1 years, and the duration of work experience was 16.2 years. The results of the qualitative phase showed 63 codes categorized in 8 subthemes and 3 themes (Table 1). The extracted concept for gender‐sensitive STIs/HIV prevention services was: “the services with the appropriate structure, including gender‐sensitive personnel, facilities, and management as well as the appropriate process, including gender‐sensitive care and education. To achieve gender‐sensitive services, appropriate policies, the collaboration of different systems, and community empowerment are necessary for development and promotion of gender‐sensitive” (Figure 2).

**Table 1:** The final version of the questionnaire to assess needs for gender‐sensitive STIs/HIV prevention services (GSPS)

	Not at all	Average	Completely
How important are following supportive policies for the gender‐sensitive STIs/HIV care services			
1. Making spouses aware of STIs for improving family health			
2. Planning for abstinence promotion and the risk reduction policy (condom use and limiting partners)			
3. Overcoming the taboos for sexual health education			
4. Promoting communication skills education to reduce high‐risk sexual behaviors			
5. Development of a comprehensive reproductive health promotion program			
6. Control and monitoring of media for advertising high‐risk sexual behaviors			
7. Promoting sexual and reproductive rights and health			
8. Facilities for providing the services for sexually active single women			
9. Facilities for providing the services for sexually active boys			
10. Overcoming the regulation barriers for declaration of high‐risk behavior in the health system			
How important are the following intersectional collaborations for the gender‐sensitive STIs/HIV care services			
1. Academic research about sexual behaviors in the community			
2. Determining common sexual medications, effective in preventing or spreading STIs			
3. Police training on appropriate reactions with women after sexual assault			
4. Integrating STIs/HIV prevention program for men, in occupational medicine			
5. Cooperating of sports organization for education and promotion of STIs/HIV prevention programs			
6. Overcoming the barriers for education about STIs/HIV prevention in schools			
How important are the following strategies for community capacitation in helping STIs/HIV prevention programs?			
1. Parents' education for promoting adolescents sexual health			
2. Promoting culturally appropriate programs for preventing sexual high‐risk behavior			
3. Collaboration of ministry of education and ministry of health in education boys with high‐risk sexual behaviors			
4. Training of teachers about gender stereotypes that make adolescents vulnerable to sexual high‐risk behaviors			
How important are the following characteristics for providers of STIs/HIV prevention services?			
1. Trained personnel about reproductive rights of women and men			
2. Trained male personnel about counseling, diagnosis, and treatment of men's STIs			
3. Trained personnel for women's counseling about self‐protection			
4. Trained personnel for educating couples about talking about sexual problems			
5. Trained personnel for providing ethical and respectful services			
6. Trained personnel for counseling about prevention of high‐risk sexual behavior originating from patriarchal stereotypes			
7. Providing care with no bias			
8. Skillful personnel in counseling couples about reducing the risk of STIs/HIV			
9. Trained personnel about laws for protecting sexually abused women			
10. Trained personnel about Islamic perceptions about reproductive rights and correcting misconceptions			
11. Skillful personnel in sexual health counseling			
12. Personnel with no misconception about women care services			
13. Trained personnel about reducing sexual risks among adolescents			
How important are the following facilities for providing STIs/HIV prevention services?			
1. A private and secure environment for STIs risk assessment			
2. Referral system for diagnosis, treatment, and follow‐up of men with STIs			
3. STIs' screening facilities for premarital couples			
4. Hotlines for response to the questions about STIs/HIV			
5. Friendly services for providing sexual health counseling to sexually active boys			
6. Friendly services for providing STIs/HIV prevention and treatment for sexually active girls			
7. Providing STIs/HIV prevention and treatment services for temporary marriage clients			
How important are the following management actions for providing STIs/HIV prevention services?			
1. Providing continuing education for STIs/HIV care providers			
2. Training of providers and managers about gender‐sensitive services			
3. Development of guidelines for providing care for sexual violence and high‐risk behaviors			
4. Development of guidelines for advocating reproductive rights of clients			
5. Development of indexes for monitoring and evaluation of gender‐sensitive services			
6. Data gathering about the high‐risk sexual behavior of adolescents			
7. Personnel protection in the case of support for women's reproductive health			
8. Solving problems of men's STIs/HIV care providers			
How important are the following care services for providing STIs/HIV prevention services?			
1. Recommending condom use to clients with high‐risk sexual behavior			
2. Assessment of penis abnormalities as the barrier for condom use			
3. Providing female condoms for women protection if necessary			
4. Providing self‐care education and the related booklets			
5. Screening of STIs among men when their wives are using IUD			
6. Women's education about the signs and symptoms of STIs in men			
7. STIs/HIV screening of men as a prenatal care			
8. STIs/HIV prevention care and counseling for premarital couples			
How important are the following educations for providing STIs/HIV prevention services?			
1. Training of women about self‐protection			
2. Sexual health education based on the men's and women's special needs			
3. Education of negotiation skills about condom use			
4. Education about reproductive rights for clients with high‐risk sexual behaviors			
5. Providing counseling with no bias and stigmatization			
6. gender‐based education of adolescents about high‐risk sexual behavior			
7. Educating clients about ways for recognizing individuals with high‐risk sexual behaviors			

Abbreviations: IUD, intrauterine device; STIs/HIV, sexually transmitted infections/human immunodeficiency virus.

### 3.2 Phase 2: The questionnaire development and psychometric properties assessment

#### 3.2.1 The questionnaire development

*The qualitative phase for the items generation*: Sixty‐three extracted codes from the qualitative phase were categorized into eight subthemes and three themes. These codes and subthemes were used for the items generation and subscales of the questionnaire development.

3.2.2

*The literature review for completing the items*: Three items were added to the item pool after the literature review, including: “Cooperating of sports organization for education and promotion of STIs/HIV prevention programs,” “Hotlines for response to the questions about STIs/HIV,” and “Providing self‐care education and the related booklets.”^45^, ^46^, ^47^ Therefore, the primary GSPS questionnaire was developed with 66 items within eight subscales. Then, the psychometric properties of GSPS were assessed.

#### 3.2.3 Psychometric properties of the questionnaire

The questionnaire was assessed regarding its validity and reliability as below:

3.2.4

*Validity of the questionnaire*: Face and content validity as the theoretical and representational validity of the questionnaire was evaluated by qualitative and quantitative methods.

*Face validity*: Qualitative face validity resulted in corrections of five items and elimination of the Item “Develop long‐term health plans to increase the participation of men and adolescent boys in the prevention of sexually transmitted diseases” with an impact score of less than 1.5.

*Content validity*: Qualitative content validity assessment resulted in corrections of three items. Two further items including the items “Planning to deal with gender taboos such as premarital sex or homosexuality” and “Eliminate criminal offenses for exposing certain sexual behaviors” were omitted because of the CVI of less than 0.7. Content validity of GSPS was confirmed by S‐CVI and S‐CVR = 0.98 and S‐CVR = 0.87, respectively.

3.2.5

*Reliability of the questionnaire*: To assess the reliability of the questionnaire, internal consistency and stability of the questionnaire were evaluated.

3.2.6

*Internal consistency*: The internal consistency of GSPS was confirmed by *α* Cronbach = 0.972.

3.2.7

*Stability*: Stability of the questionnaire was assessed and showed by test–retest method and calculating the intracluster correlation coefficient = 0.910.

**Figure 3:**
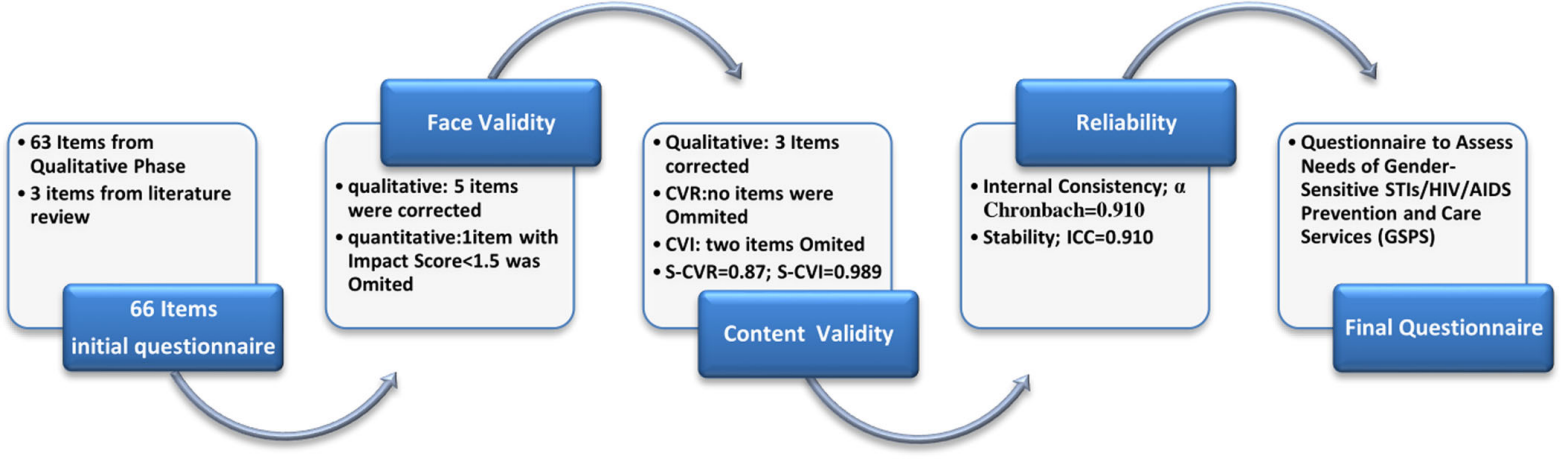
Results of validity and reliability assessment process of questionnaire to assess needs of gender‐sensitive STIs/HIV prevention and care services (GSPS‐63). AIDS, acquired immunodeficiency syndrome; ICC, intraclass correlation coefficient; S‐CVI, Scale‐level content validity index; S‐CVR, scale‐level content validity ratio; STIs/HIV, sexually transmitted infections/human immunodeficiency virus

After the procedure of face, content, reliability assessment, and the necessary corrections, the final valid and reliable questionnaire was developed with 63 items. Figure 3 shows the procedure of designing and validating the process of GSPS.

3.2.8

*Describing the questionnaire and the scoring system*: GSPS was a self‐completed questionnaire with 63 items. The items were scored on a three‐point scale of 0–2, for the importance of the item from “not at all” to “Completely.” The scores of items were summed up for calculation of total scores and then calculated scores were converted to 0–100. To convert the scores of the subscales and the entire questionnaire to a score of 0–100, the following conversion formula was used. Adjusted score = (the raw score obtained − minimum possible score/maximum possible − minimum possible score) × 100. High scores demonstrate the more important needs as the priorities for gender‐sensitive STIs/prevention services.

### 3.3 Phase 3: The quantitative phase of the study “assessment needs for gender‐sensitive STIs/HIV prevention services”

Two hundred ninety health providers participated in the study, with an average age of 33.48 ± 6.53 years (mean ± SD) and a working experience of 9.74 ± 5.65 years. The response rates of the participants were 100%. Table 2 demonstrated the participants' demographic characteristics.

**Table 2:** Demographic characteristics of the STIs/HIV health care providers in Shiraz 2016 (*n* = 290)

Characteristics	Category	Number	%
Gender			
Female		275	94.8
Male		15	5.2
Age			
20–30		129	44.5
31–40		121	41.7
>40		40	13.8
Education			
Midwife (bachelor)		229	79.0
Health educator (bachelor)		23	10.8
Midwife (graduate diploma)		30	7.9
Midwife (master)		8	2.8
Job experience (years)			
2–10		179	61.7
11–20		104	35.9
>20		7	2.3

Abbreviation: STIs/HIV, sexually transmitted infections/human immunodeficiency virus.

Results of the quantitative descriptive phase demonstrated the highest priorities for the gender‐sensitive process (including care and educational dimensions) and then gender‐sensitive structure. The finding showed the highest priorities for the gender‐sensitive educational (92.01 ± 11.76%) and care services (92.11 ± 12.04%), respectively. Table 3 shows mean scores of all eight dimensions of the services and Table 4 demonstrates two priorities for the eight dimensions of the services.

**Table 3:** Needs for gender‐sensitive STIs/HIV prevention services based on their priorities

Sections	Dimensions	Mean ± SD (score 0–100)	Mean ± SD (score 0–100)
Process	Education	92.01 ± 11.76	92.06 ± 12.04
	Care	92.11 ± 12.04	
Structure of the Services	Human resources	91.15 ± 13.31	90.52 ± 12.04
	Management	90.53 ± 13.30	
	Facilities	89.35 ± 15.99	
Gender‐sensitive policies	Supportive policies	86.22 ± 16.31	87.24 ± 14.05
	Intersectional cooperation	86.80 ± 17.36	
	Community empowerment	91.07 ± 14.09	

Abbreviation: STIs/HIV, sexually transmitted infections/human immunodeficiency virus.

**Table 4:** Items with the highest priorities in each dimension of STIs/HIV prevention services

Dimensions	Priorities	Score		Frequency (%)		
		Mean(0–2)	SD	Not at all: 0	Roughly: 1	Completely: 2
Care	Recommending condom use to clients with high‐risk sexual behavior	1.88	0.31	0	11.0	89.0
	Assessment of penis abnormalities as the barrier for condom use	1.88	0.31	0	11.4	88.6
Education	Educating women about self‐protection	1.87	0.33	0	12.8	87.2
	Sexual health education based on the men's and women's special needs	1.86	0.35	0.3	12.8	86.9
	Education of negotiation skills	1.86	0.34	0	13.8	86.2
Personnel	Trained providers about reproductive rights of women and men	1.86	0.34	0	13.8	86.2
	Trained male personnel about counseling, diagnosis, and treatment of men's STIs	1.85	0.35	0.3	13.4	86.3
Management	Providing continuing education for STIs/HIV care providers	1.84	0.35	0	15.2	84.8
	Personnel protection in the case of support for women's reproductive health	1.82	0.42	1.7	13.8	84.5
Facilities	Providing a secure and private environment for STIs risk assessment	1.84	0.38	0.7	14.5	84.8
	Introducing facilities for diagnosis, treatment, and follow‐up of men with STIs	1.83	0.39	0.7	16.6	82.7
Supportive policies	Making spouses aware of STIs for improving family health	1.80	0.4	0.7	18.6	80.7
	Planning for abstinence promotion and the risk reduction policy (condom use and lowering partners)	1.79	0.47	3.1	14.1	82.8
Intersectional cooperation	Academic research about sexual behavior in different communities	1.75	0.46	1.7	20.7	77.6
	Determining common sexual medications in the private sector, effective in preventing or spreading STIs	1.75	0.48	2.4	19.7	77.9
Community capacitation	Parents education for improving adolescents' sexual health	1.83	0.37	0	16.9	83.1
	Promoting culturally appropriate programs for preventing sexual high‐risk behavior	1.82	0.31	1.0	15.9	83.1

Abbreviation: STIs/HIV, sexually transmitted infections/human immunodeficiency virus.

## 4 DISCUSSION

This is the first mixed exploratory study to explain the concept and dimensions of gender‐sensitive STIs/HIV/AIDS prevention services and then the needs and priorities for providing these services.

Gender‐sensitive STI/HIV prevention services were defined as: “the services with the appropriate structure, including gender‐sensitive personnel, facilities, and management, as well as the appropriate process, including gender‐sensitive care and education. To achieve gender‐sensitive services, appropriate policies, the collaboration of different systems, and community empowerment are necessary for development and promotion of gender‐sensitive.” According to the Donabdian model for evaluating the quality of health care,^48^ information about the quality of care can be drawn from three dimensions of services, including “structure,” “process,” and “outcomes.” Assessment of the gender sensitivity of the services mentioned the importance of gender sensitivity of process and structure of STIs/HIV services. Consistent with other studies, the present study emphasizes the importance of cultural factors that define gender roles in any community on the quality of the services.^49^

Subsequently, results of the quantitative phase showed that procedures of the services, (both care and educational process) are the most important priorities in providing these services. The most important care was stated to be “Recommending condom use to clients with high‐risk sexual behavior” and then “assessment of penis abnormalities as a barrier for condom use.” Otherwise, the results emphasized overcoming gender‐based barriers to men's sexual health and promoting condom use. Diminishing taboos and providing appropriate counseling for risk reduction and condom use as well as assessment of male reproductive system are also emphasized in previous studies.^50^

Results showed three priorities for educational procedures, including “women's education about self‐protection” and “Sexual health education based on the men's and women's special needs” and “education on negotiation skills about condom use.” Women's education about these areas helps to improve couples' relationships, couples' sexual health, preventing high‐risk behavior of spouses, such as spouses' out of marriage partnership and multiple sexual partnerships and preventing sexual abuse, and finally, empower women's sexual reproductive health. It is documented that knowledge about condom and condom accessibility is not enough, rather overcoming the barriers of condom use are necessary, such as the overwhelming unequal status of women and men in a community and masculinity and myth about the male role in decision‐making. Furthermore, women's education on negotiation skills can be effective on the promotion of condom use.^7^

The gender‐sensitive structure of the services showed the need for gender‐sensitive providers, facilities, and management. In the quantitative assessment, two priorities were demonstrated including “Trained Providers about reproductive rights of women and men” and “Trained male personnel about counseling, diagnosis, and treatment of men's STIs.” Female providers in many health care systems are the barriers for attending men in giving any care services, especially for STIs prevention and treatment services. Besides male care providers of some health care services lack the necessary information, which is also another barrier for attending men for the care and treatment of STIs.^51^

Results revealed two priorities for gender‐sensitive management of the services, consisting of needs for “Providing continuing education for STIs/HIV care providers” and “Personnel protection in the case of support for women's reproductive health". World Health Organization^52^ emphasized on development of health providers' education about reproductive health as a top priority in the management of health systems and also mentioned to be important for combating STIs dissemination. Besides, the need for “protecting personnel in the case of support of women's reproductive health right” is also demonstrated in previous studies. In these studies, health providers emphasized the necessity for personnel's education about appropriate interactions as well as the need for developing the legal supportive systems for this personnel in health and public organizations.^53^, ^54^

The results showed two top priorities for reforming facilities of the services, including “Providing a secure and private environment for STIs risk assessment” and “Introducing facilities for diagnosis, treatment, and follow‐up of men with STIs.” It is demonstrated that men need private counseling and care services at an appropriate time, preferably in the evening or weekend.^24^, ^55^ Furthermore, the services should be comprehensive and contain STIs/HIV preventive as well as curative care and follow‐up facilities.^47^

The results demonstrated “supportive policies” as the priority of the services. Two items showed the highest scores, including “Making spouses aware about STIs, for improving family health” and “Planning for abstinence promotion and the risk reduction policy.” It is demonstrated that spouses' awareness about STIs prevention, their treatment, and preventing from recurrent infections is important.^56^ In many countries spouses' counseling is compulsory in health services and counseling is performed in STI cases.^57^ It seems a comprehensive sexual health education including a risk reduction program should be considered as a supportive policy for our community. Planning of skill‐based educational interventions that enhance behavioral and preventive beliefs and promote abstinence would reduce the risk of STIs.^58^

Findings also showed two priorities for the intersectional cooperation for supporting the gender‐sensitive services, including “Academic research about sexual behavior in different communities” and “Determining common sexual medications in the private sector, effective on preventing or spreading of STIs.” Extensive research was demonstrated as an essential base for an evidence‐based intervention for promoting sexual health.^59^ Since there are some limitations in public sectors for providing services to high‐risk individuals, information about taking these medications is low. It is revealed that a minority of youth are involved in premarital relationships.^60^ Homosexuality is also forbidden and there is no information about the frequency of AIDS among these high‐risk groups.^61^ Therefore, there is the global problem that information about high‐risk groups and social outcast groups such as sex workers are limited and it is the reason for limited success in STIs/HIV programs and so academic researchers are necessary.^62^

Priorities for community capacitation, as another gender‐sensitive policy, were “Parents education for improving adolescents' sexual health” and “Promoting of culturally appropriate programs for preventing sexual high‐risk behavior.” Several studies documented that a close relationship between parents and children can reduce high‐risk behaviors^63^, ^64^, ^65^ and prevent STIs/HIV among adolescents.^66^, ^67^ Studies demonstrated a majority of parents are unwilling to encourage their sons about condom use, as they presume youth are not sexually active. Thus developing educational programs for parents are necessary.^68^, ^69^ Besides, the educational level of parents was positively associated with talking of parents and adolescents about sexual health.^70^ Also, in many Moslem countries, the success of high‐risk prevention programs is imitated because of the stigmatization of high‐risk sexual behaviors, while homosexuality or out of marriage sexual relationships and sex working is common in these countries and needs more care and risk reduction counseling services.^71^ Many studies are also emphasized women's empowerment in sexual reproductive health behaviors as an essential policy for preventing STI/HIV.^72^, ^73^ Community empowerment is also necessary for moderating the effects of gender on STI risk‐related sexual behavior by mass media cooperation.^74^

In this mix sequential exploratory study, we tried to assess the need for promoting gender‐based STI/HIV/AIDS prevention services in Iran, with a valid and reliable tool. Since gender stereotypes in Iranian culture are somewhat similar to other Middle‐East countries, the needs shown in this study can be similar for the countries of this region. Besides, the methodology of this study can be applied not only in the region but also in all countries to identify the specific needs of men and women for providing the appropriate gender‐based services and so to improve the quality of the services. According to The United Nations Population Fund's research report on the effects of culture and religion on reproductive health that was performed in 169 countries around the world, gender stereotypes were mentioned as the main barrier for reproductive health promotion. Then further research was recommended to identify needs in other countries with different cultures, to provide evidence‐based strategies to promote reproductive health by removing cultural barriers.^75^

Therefore, we emphasize that gender‐restrictive norms undermine the health and wellbeing of all people. It is essential to address gender bias in the health system for achieving Sustainable Development Goals. Also, the World Health Organization's Global Health Sector Strategy on STIs 2016–2021 highlights the importance of gender equality in STI prevention and control programs.^76^ Given the global recommendations and recent momentum in incorporating gender equality in policies and programs, the finding of the present study certainly has positive implications for the delivery of gender‐sensitive STIs/HIV preventive services. Also, this innovative design can be replicated in other settings. To our knowledge, this is the first study for assessing the needs for gender‐sensitive services, with an innovative design in the region where gender stereotypes are usually highlighted. We recommend conducting similar studies in other areas of reproductive health and other countries of the region and the world, especially in countries with deeper gender equality gaps.

### 4.1 Limitation

The taboo of talking about sexual issues was a limitation that was controlled by an explanation about the confidentiality of the information by providing a nameless questionnaire. Also, we did not mention the needs of individuals with gender dysphoria, as we believe they have vast special needs. Besides, since homosexuality in Iran is against the law and sharia, researching this area requires a different research design. We recommend further research focusing on sexual orientation/behavior and the needs for providing the services based on the specific needs of individuals with opposite sexual orientation and behavior. It should be noted that people with dysphonia have permission for surgery and gender reassignment.^77^

Since the aim of the present study was to explain and determine the needs of the system to provide gender‐sensitive STI/HIV prevention services, we develop and use GSPS questionnaire with theoretical validity (content and face validity). We suggest factor analysis for empirical construct and criterion validity as well as theoretical validity assessment in future studies that aim at tool development.

## 5 CONCLUSION

This was the first mixed exploratory study to assess needs for gender‐sensitive STIs/HIV prevention services. With a theoretical valid and reliable tool (GSPS with S‐CVI = 0.98; S‐CVR = 0.87; *α* Cronbach = 0.91 stability; ICC = 0.910). It was demonstrated that providing STIs/HIV prevention services requires reforms to achieve a gender‐appropriate educational and care process, as well as a gender‐sensitive structure, including gender‐sensitive personnel, facilities, and management. These dimensions need advocacy by supportive policies and intersectional cooperation, as well as equipping the community for promoting gender‐sensitive STIs/HIV prevention services. Certainly, providing these comprehensive and efficient services improves the quality of the STIs/HIV prevention services. In this article, the need and priorities for making the gender‐sensitive STIs/HIV prevention services were explained. A valid and reliable questionnaire to assess these needs was developed and introduced. The paper made evidence‐based suggestions for managers and policymakers to improve the quality of the services by providing gender‐sensitive services.

## CONFLICTS OF INTEREST

The authors declare no conflicts of interest.

## AUTHOR CONTRIBUTIONS

*Conceptualization*: Masoumeh Simbar, Fatemeh Rahmanian, Soheila Nazarpour, Ali Ramezankhani. *Data curation*: Masoumeh Simbar, Fatemeh Rahmanian, Farid Zayeri. *Formal analysis*: Fatemeh Rahmanian, Farid Zayeri: Not applicable. *Investigation*: Masoumeh Simbar, Fatemeh Rahmanian, Soheila Nazarpour, Ali Ramezankhani, Farid Zayeri. *Methodology*: Masoumeh Simbar, Fatemeh Rahmanian, Ali Ramezankhani. *Project Administration*: Masoumeh Simbar, Ali Ramezankhani. *Resources*: Masoumeh Simbar, Fatemeh Rahmanian, Soheila Nazarpour. *Software*: Not applicable. *Supervision*: Masoumeh Simbar, Soheila Nazarpour, Ali Ramezankhani, Farid Zayeri. *Validation*: Masoumeh Simbar, Fatemeh Rahmanian, Farid Zayeri. *Visualization*: Not applicable. *Writing—original draft preparation*: Masoumeh Simbar, Soheila Nazarpour. *Writing—review and editing*: Masoumeh Simbar, Soheila Nazarpour. All authors have read and approved the final version of the manuscript.

## Data Availability

The corresponding author Soheila Nazarpour confirms that he had full access to all of the data in the study and takes complete responsibility for the integrity of the data and the accuracy of the data analysis.
